# Analysis of predictors of clinical pregnancy and live birth in patients with RIF treated with IVF-ET technology: a cohort study based on a propensity score approach

**DOI:** 10.3389/fmed.2024.1348733

**Published:** 2024-04-16

**Authors:** Yan Jia, Zhonghua Ai, Xianglong Zhu, Zhuohang Che, Adhikari Pratikshya, Songyuan Tang, Qiong Zhang

**Affiliations:** ^1^Department of Reproductive Immunology, Sichuan Jinxin Xi’nan Women’s and Children’s Hospital, Chengdu, Sichuan, China; ^2^Acupuncture and Tuina School, Chengdu University of Traditional Chinese Medicine, Chengdu, Sichuan, China; ^3^Chengdu Jinjiang District Maternal and Child Health Hospital, Chengdu, Sichuan, China; ^4^Institute of Health Studies, School of Public Health, Kunming Medical University, Kunming, Yunnan, China; ^5^Department of Reproductive Medicine, The Affiliated Hospital of Yunnan University, Kunming, Yunnan, China

**Keywords:** *in vitro* fertilization, recurrent embryo implantation failure, clinical pregnancy, live birth, predictor

## Abstract

**Objective:**

To investigate the predictors of clinical pregnancy and live birth rate in patients with recurrent embryo implantation failure (RIF) treated with *in vitro* fertilization-embryo transfer (IVF-ET) technique.

**Method:**

This retrospective cohort study was conducted in Jinjiang District Maternal and Child Health Hospital, Chengdu City, Sichuan Province, China. Patients were recruited who were enrolled at this hospital between November 1, 2019 and August 31, 2022, and who met the following criteria: a frozen embryo transfer (FET) at day 5 or 6 blastocyst stage was performed and the number of transfer cycles was not less than two. We collected information on age, height, weight, number of embryo transfer cycles, and information related to clinical outcomes. We used the group of patients who underwent ERA testing as the study group and those who underwent FET only as the control group, and matched baseline characteristics between the two groups by propensity score to make them comparable. We compared the differences in clinical outcomes between the two groups and further explored predictors of pregnancy and live birth using survival analysis and COX regression modeling.

**Results:**

The success rate of clinical pregnancy in RIF patients was 50.74% and the live birth rate was 33.09%. Patients in the FET group were less likely to achieve clinical pregnancy compared to the ERA group (*HR* = 0.788, 95%*CI* 0.593–0.978, *p* < 0.05). Patients with >3 previous implantation failures had a lower probability of achieving a clinical pregnancy (*HR* = 0.058, 95%*CI* 0.026–0.128, *p* < 0.05) and a lower likelihood of a live birth (*HR* = 0.055, 95%*CI* 0.019–0.160, *p* < 0.05), compared to patients with ≤3 previous implantation failures. Patients who had two embryos transferred were more likely to achieve a clinical pregnancy (*HR* = 1.357, 95%*CI* 1.079–1.889, *p* < 0.05) and a higher likelihood of a live birth (*HR* = 1.845, 95%*CI* 1.170–2.910, *p* < 0.05) than patients who had a single embryo transfer. Patients with concomitant high-quality embryo transfer were more likely to achieve a clinical pregnancy compared to those without high-quality embryo transfer (*HR* = 1.917, 95%*CI* 1.225–1.863, *p* < 0.05).

**Conclusion:**

Not receiving an ERA, having >3 previous implantation failures, using single embryo transfer and not transferring quality embryos are predictors for clinical pregnancy in patients with RIF. Having>3 previous implantation failures and using single embryo transfer were predictors for live birth in patients with RIF.

## Introduction

With the rapid development of society, changes in people’s lifestyles, deteriorating living environments, as well as increased social pressure and artificial termination of pregnancy, infertility has become the third most prevalent human disease after cancer and cardiovascular diseases (1). Infertility is defined as the failure of a couple to achieve a pregnancy within 1 year without contraception and with regular sexual intercourse (2). Infertility affects 5 to 8% of couples in developed countries and up to 30% in developing countries (3). In China, no fewer than 50 million women suffer from infertility, accounting for about 15% of women of childbearing age (4). Assisted Reproductive Technology (ART) offers hope to families desiring a new life, and includes Artificial Insemination, *In Vitro* Fertilization - Embryo Transfer (IVF-ET) and a range of techniques derived from it. IVF-ET refers to the process of combining female eggs and male sperm in an external laboratory setting to form fertilized embryos, which are then transferred into the female uterus to facilitate pregnancy and childbirth. IVF-ET is a relatively recent but rapid development research technique (5).

Embryo implantation is the process of transferring multiple early, well-developed blastocysts that have undergone *in vitro* fertilization or other *in vitro* fertilization techniques into the uterine cavity of a woman for the purpose of implantation and pregnancy. In assisted reproduction, Recurrent Embryo Implantation Failure(RIF) is defined as the failure to achieve pregnancy after multiple cycles of assisted reproductive technology and the transfer of multiple high-quality blastocysts. RIF remains the speed-limiting step in ART. Many researchers have used the observation of an intrauterine gestational sac on ultrasound as a criterion for implantation success or failure when describing RIF (6, 7). Endometrial Receptivity Analysis (ERA) is a genetic diagnostic method based on transcriptional genomics, which analyses the expression of 248 genes associated with the window of implantation, allowing a more accurate assessment of the endometrial window of implantation status and finding the right “window of implantation” (WOI) for the woman undergoing treatment (8). “It is an important tool in the treatment of recurrent embryo implantation failure (8). To the best of our knowledge, there is a lack of relevant studies on pregnancy and live birth predictors in the RIF population in China. Therefore, the aim of this study was to investigate the predictors affecting pregnancy and live birth in the Chinese RIF population and to provide a basis for relevant institutions and personnel to develop effective strategies.

## Methods

### Study subject

This study was a retrospective cohort study that patients with RIF who received infertility treatment at Jinjiang District Maternal and Child Health Hospital in Chengdu City between November 1, 2019 and August 31, 2022 were selected for this study and collected information related to age, height, weight, embryo transfer cycle, and clinical outcomes of these patients. For patients undergoing endometrial biopsy, written informed consent was obtained from the patients prior to treatment for the evaluation of endometrial receptivity. Inclusion criteria: age between 18 and 45 years; BMI between 18.5 and 30 kg/m^2^; couples experiencing multiple embryo implantation failures (at least 2 cycles of embryo transfer, or transfer of at least 3 quality blastocysts with a Gardner score of 4BB or higher) (9). Exclusion criteria: patients with genetic disorders, anatomical abnormalities of the reproductive tract, infections; patients with more severe abnormal semen quality or weak spermatozoa in the male partner. Ultrasound-confirmed uterine malformations, abnormal karyotype, hormonal or metabolic disorders and known clinical autoimmune disorders. Patients with incomplete documentation of relevant clinical outcomes such as pregnancy and delivery.

The patients included in this study were all patients with recurrent embryo implantation failure (RIF). 239 patients in the ERA group and 513 patients in the FET group were included in the study who met the criteria before propensity score matching. A caliper matching method was used, with a caliper value of 0.02 and a matching ratio of 1:1. Ultimately, the ERA and FET groups were successfully matched to 204 pairs. Among them, there were 207 cases in the successful pregnancy group and 201 cases in the failed pregnancy group; 135 successful live births group and 273 failed live births group.

### Endometrial sampling and processing

Patients in the ERA group received personalized embryo transfer (PET), while those in the FET group received only conventional frozen embryo transfer. Patients in the ERA group underwent ERA testing under hormone replacement therapy (HRT) cycles or natural cycles (10). When performing ERA testing, the process consists of several steps. First, researchers identify genes using Agilent array technology, a step that provides the basic data for subsequent analyses. Next, microarray chips are customized for specific research needs to ensure efficient monitoring and detection of key genes. Subsequently, the resulting bioinformatics data were collected and analyzed, and through systematic interpretation and analysis of these data, relevant features and changes in the endometrium were revealed. Finally, gene classification techniques were used to accurately assess and classify endometrial tolerance (10). In the HRT cycle, 300 mg of progesterone soft gels Utrogestan (CYNDEA PHARMA SL; Olvega, Spain) were taken every 12 h at the beginning for 120 ± 3 h. A sterile pipette (Jiaobao Healthcare Technologies Ltd., China) was used to collect 50 to 70 mg of endometrial biopsy samples from the base of the uterus on Dydrogesterone tablets (dydrogesterone; Abbott Biologics Ltd.; Amstelveen, The Netherlands) were administered twice daily (P + 5) 20 mg on day 5 after the start of menstruation. Endometrial specimens were sampled 7 days after the luteinizing hormone surge (LH + 7) or 7 days before the administration of human chorionic gonadotropin (hCG + 7) during the natural cycle. All endometrial specimens were transferred to frozen tubes (Biosigma S.p.A.; Kona, Italy) containing 1.5 mL of RNA late solution (Qiagen GmbH; Hilden, Germany) and shaken vigorously to stabilize the genetic material in the tissue. Endometrial specimens were kept at 4°C for at least 4 h or stored at −20°C and then shipped at room temperature for final ERA testing. Patients in the FET group did not receive ERA but underwent transfer of embryos 120 to 126 h after luteal transformation.

### Variable selection

Predictors of clinical pregnancy and live birth in RIF patients treated with ART were set as follows.

**Dependent variable**: IVF-ET clinical pregnancy success, IVF-ET clinical failure; IVF-ET live birth successful, IVF-ET live birth failure.

**Independent variable**: Variable setting: 0 for ERA, 1 for FET; 0 for age < 35 years, 1 for age ≥ 35 years; 0 for BMI <24 kg/m^2^, 1 for ≥24 kg/m^2^; 0 for sinus follicle count (AFC) ≥4, 1 for <4; 0 for anti-Müllerian testicular hormone (AMH) ≥2, 1 for <2; 0 for follicle stimulating hormone (FSH) ≥1.7, 1 for 1 for less than 1.7; 0 for ≤3 previous implantation failures, 1 for >3 previous implantation failures; 0 for normal endometrial thickness, 1 for abnormal; 0 for single embryo transfer, 1 for transfer of two embryos; 0 for no quality embryos transfer, 1 for quality embryos transfer.

**Clinical pregnancy criteria**: first pelvic ultrasound performed 30 to 35 days after embryo transfer, with at least one gestational sac and primitive cardiac pulsation detected in the uterine cavity and no gestational sac outside the uterus.

**Miscarriage criteria**: Miscarriage after ART can be defined as spontaneous termination of pregnancy throughout the gestational cycle, including embryo abortion and stillbirth. Biochemical pregnancy failure is defined as positive serum hCG 2 weeks after embryo transfer and no gestational sac detected on ultrasound 1 month later. Biochemical pregnancies, spontaneous abortions and ectopic pregnancies are all considered pregnancy failures.

**Live birth criteria**: A live birth is defined as a product of conception that is able to breathe or shows other evidence of life, such as heartbeat, respiration, and umbilical cord arterial pulsation, after it has completely left the mother’s body, regardless of the duration of the gestation, and is considered to be a live birth.

### Statistical analysis

The Kolmogorov–Smirnov test was used to test the distribution of continuous variables. All measurements were expressed as mean ± standard deviation (SD) or quartiles, median, and all categorical data were described as numbers and percentages. Comparisons of measures were made using the t-test or Wilcoxon ran-sum test, and categorical data were tested using the chi-square test. Survival analysis was performed using the Kaplan–Meier method, using the number of transplant cycles as the unit of analysis. In the survival analysis, the cumulative clinical pregnancy rate and live birth rate were compared using the Logrank test. Multivariate analysis was performed using COX proportional risk models. *p*-values <0.05 were considered statistically significant. All statistical analyses were performed using the statistical software R language version 4.2.3.

## Results

### Basic information of the patients

After propensity scores were matched at a ratio of 1:1, the ERA and control groups were matched to 204 cases each, and a balanced comparison between the two groups showed no statistically significant differences in all covariates (*p* > 0.05), indicating that the baseline was balanced and well matched after the matching treatment was applied to both groups. As shown in Table 1.

**Table 1:** Baseline profiles of patients in the ERA and FET groups after propensity score matching.

Features	ERA (*n* = 204)	FET (*n* = 204)	*x*^2^/*t*/*Z*	*p*-value
Female age(y)	33.53 ± 3.68	33.27 ± 4.21	0.651	0.515
BMI(kg/m^2^)	21.32 ± 2.71	21.25 ± 2.73	0.262	0.793
AFC	17	16	0.644	0.802
	(11.00,22.00)	(11.25,21.75)		
AMH(ng/ml)	3.51	3.78	1.040	0.230
	(2.41,5.61)	(2.77,5.42)		
FSH(IU)	7.22	7.48	0.891	0.405
	(6.00,8.35)	(6.21,8.79)		
No. of previous implantation failure	2	3	1.217	0.224
	(2.00,3.00)	(2.00,3.00)		
Endometrial thickness (mm)	9.5	9	1.337	0.056
	(8.50,10.50)	(8.00,10.50)		
No. of embryos transferred, n(%)			0.041	0.840
1	82(40.2)	84(41.2)		
2	122(59.8)	120(58.8)		
Number of high quality embryos, n(%)			0.288	0.866
0	74(36.3)	69(33.8)		
1	81(39.7)	83(40.7)		
2	49(24)	52(25.5)		

AMH, Anti-Müllerian hormone; BMI, body mass index; ERA, endometrial receptivity array; FET, frozen–thawed embryo transfer; FSH, follicle stimulating hormone.

### Distribution of propensity scores for matched ERA and FET groups

**Figure 1:**
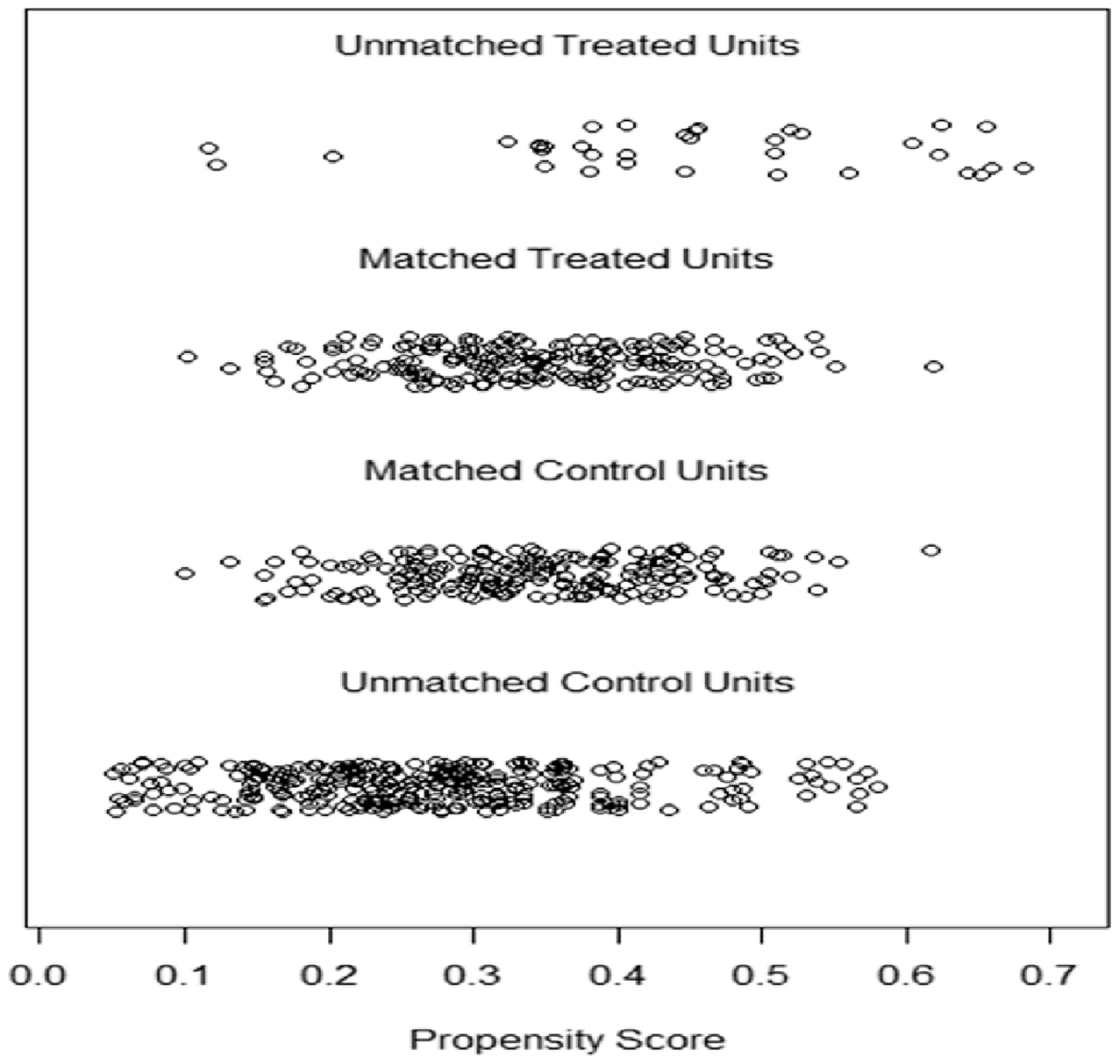
Propensity score distribution jitter plot.

Figure 1 shows the propensity score distribution jitter plot, which represents the distribution of propensity score values between the matched and unmatched patients in the ERA and FET groups (unmatched treatment units represent the unmatched ERA group, matched treatment units represent the matched ERA group). The distribution of propensity score values between matched and unmatched patients (unmatched treatment units represent the unmatched ERA group, matched treatment units represent the matched ERA group, unmatched control units represent the unmatched FET group) gives an idea of the effect of matching. The position of the dots indicates the size of the patient’s propensity score. The results in Figure 1 show that the distribution of propensity score values for the matched ERA and FET groups are relatively close to each other and the matching effect is good.

### The clinical pregnancy rate in the ERA group is constantly increasing

**Figure 2:**
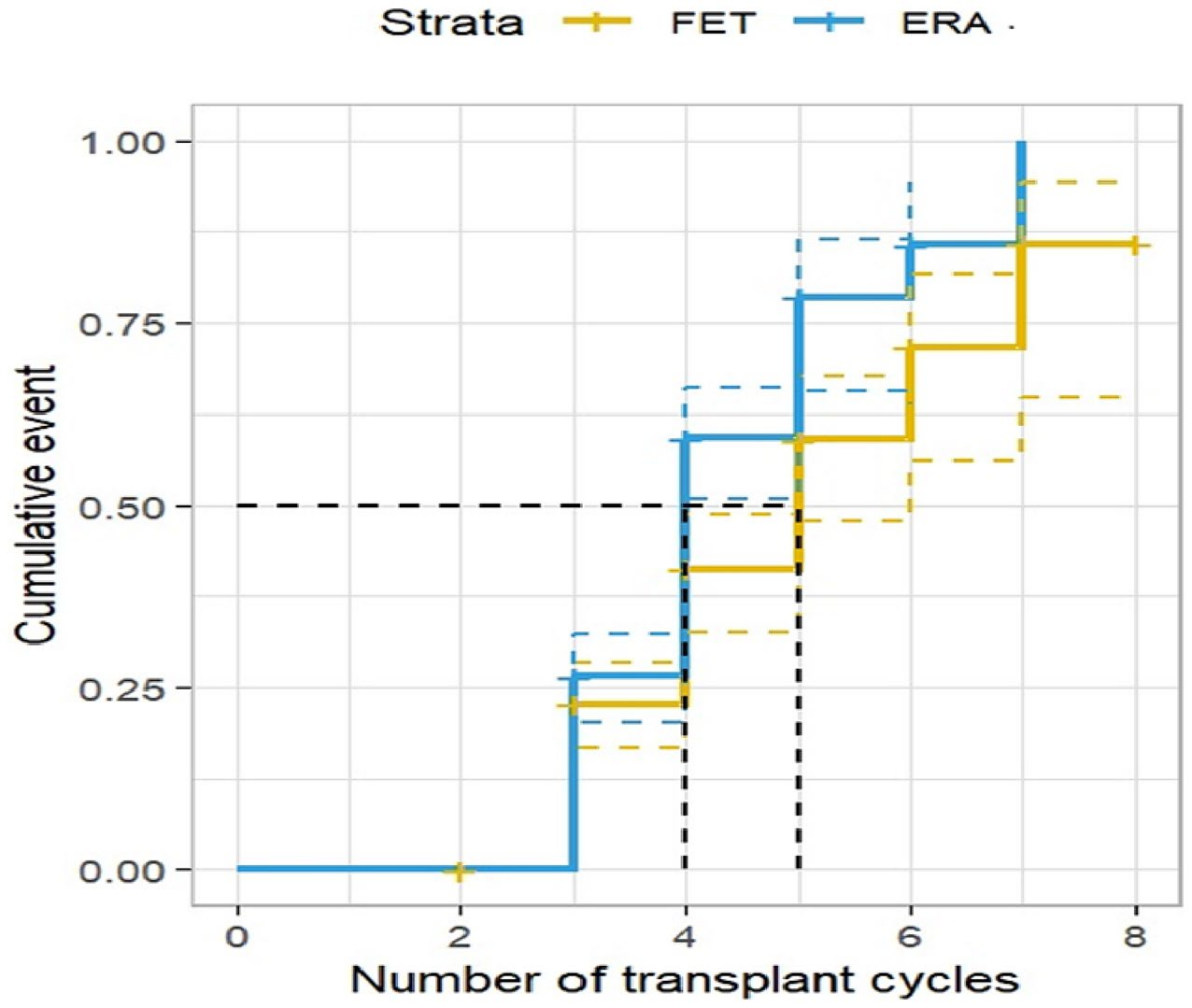
Clinical pregnancy rate by transplant cycle in the ERA and FET. The horizontal coordinate represents the number of transplant cycles and the vertical coordinate represents the clinical pregnancy rate. This figure shows that as the number of transplant cycles increased, the clinical pregnancy rate was significantly higher in the ERA group than in the FET group.

Of the 408 RIF patients investigated, 207 were diagnosed as clinically pregnant with a clinical pregnancy rate of 50.74%. The results of the survival analysis showed that the clinical pregnancy rate in the ERA group increased as the number of transfer cycles increased, with the clinical pregnancy rate in the ERA group being significantly higher than that in the FET group at all cycles, except from the third to the fourth cycle when the cumulative clinical pregnancy rate in both groups converged, and the difference was statistically significant (*p* < 0.05), as shown in Figure 2.

### Kaplan Meier analysis of factors affecting clinical pregnancy in patients with RIF

Group, Female age, BMI, Endometrial thickness and other relevant variables were analyzed using the Kaplan–Meier method. The results of the study showed that group, AMH, number of failed transfers, number of embryos transferred, and the availability of quality embryos for transfer had an impact on the patients’ clinical pregnancy, with statistically significant differences (*p* < 0.05), as detailed in Table 2.

**Table 2:** Kaplan–Meier analysis of factors influencing clinical pregnancy in patients with RIF.

Characteristics	Number of people (*n* = 408)	Number of patients pregnancy (*n* = 207)	Median	*X* ^2^	*p*-value
Group					
ERA	204	116	4	4.210	0.040
FET	204	91	4		
Female age					
<35	264	135	4	0.263	0.608
≥35	144	72	5		
BMI					
Normal	341	171	4	0.127	0.721
Overweight/Obesity	67	36	4		
AFC					
Normal	148	73	5	0.646	0.422
Anomalies	260	134	4		
AMH					
Normal	284	135	5	3.908	0.048
Anomalies	124	72	4		
FSH					
Normal	306	158	4	1.061	0.303
Anomalies	102	49	5		
No. of previous implantation failure					
≤3	346	175	4	70.517	<0.001
>3	62	32	6		
Endometrial thickness					
Normal	232	120	4	0.085	0.770
Anomalies	176	87	5		
No. of embryos transferred					
1	166	65	5	6.201	0.013
2	242	142	4		
Embryo Quality					
Cycles with high-quality embryos	265	151	4	7.491	0.006
Cycles without high-quality embryos	143	56	5		

### Analysis of factors affecting clinical pregnancy in patients with RIF using COX regression model

Table 3 analysis of factors that may influence clinical pregnancy in patients with RIF by COX regression models: patients in the FET group had a smaller chance of obtaining a clinical pregnancy compared to those in the ERA group (*HR* = 0.788, 95% *CI* 0.593–0.978, *p* < 0.05). Patients with >3 previous transfer failures were less likely to achieve a clinical pregnancy compared to patients with ≤3 previous transfer failures (*HR* = 0.058, 95% *CI* 0.026–0.128, *p* < 0.05). Patients with two embryos transferred were more likely to achieve a clinical pregnancy than those with a single embryo transfer (*HR* = 1.357, 95% *CI* 1.079–1.889, *p* < 0.05). Patients with quality embryo transfer were more likely to achieve a clinical pregnancy than those without quality embryo transfer (*HR* = 1.917, 95% *CI* 1.225–1.863, *p* < 0.05).

**Table 3:** COX regression analysis of factors influencing clinical pregnancy in patients with RIF.

Characteristics	*β*	*SE*	*wald*	*HR*	95%*CI*	*p-*value
Group						
ERA	-	-	-	1	-	-
FET	−0.238	0.146	4.67	0.788	0.593–0.978	0.049
Female age	0.021	0.02	1.062	1.021	0.982–1.062	0.303
BMI	0.015	0.026	0.316	1.015	0.964–1.069	0.574
AFC	−0.002	0.012	0.037	0.998	0.975–1.021	0.847
AMH	0.029	0.03	0.95	1.03	0.971–1.092	0.33
FSH	−0.007	0.039	0.032	0.993	0.920–1.072	0.859
No. of previous implantation failure						
≤3	-	-	-	1	-	-
>3	−2.846	0.409	48.376	0.058	0.026–0.128	<0.001
Endometrial thickness	0.036	0.042	0.736	1.037	0.954–1.127	0.391
No. of embryos transferred						
1	-	-	-	1	-	-
2	0.305	0.172	3.961	1.357	1.079–1.889	0.039
Embryo Quality						
Cycles without high-quality embryos	-	-	-	1	-	-
Cycles with high-quality embryos	0.179	0.1	3.817	1.917	1.225–1.863	0.043

### The live birth rate in the ERA group has shown a positive trend of increase

**Figure 3:**
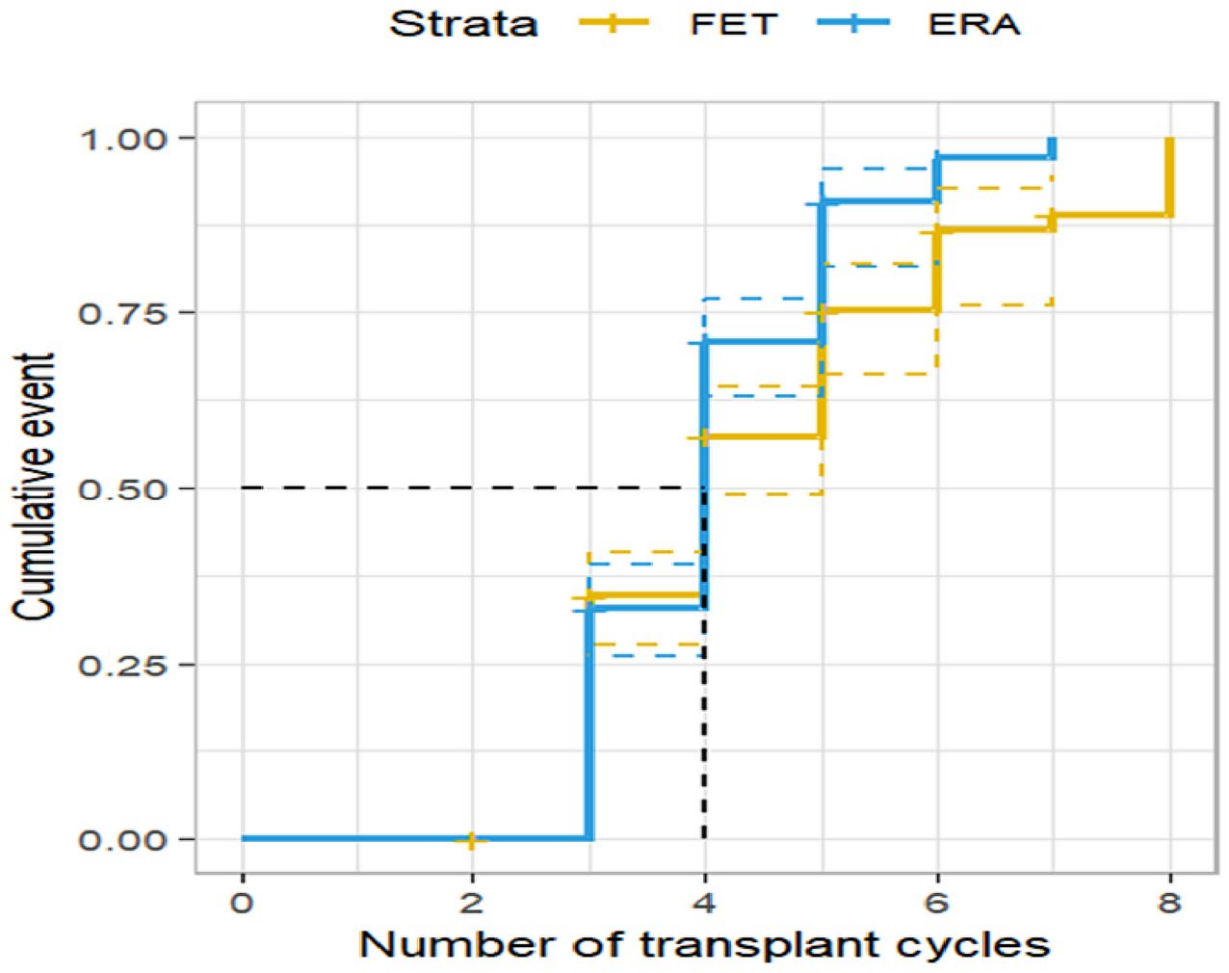
Live birth rate by transplantation cycle in ERA and FET. The horizontal coordinate represents the number of transplantation cycles and the vertical coordinate represents the live birth rate. This figure shows that as the number of transplant cycles increased, the live birth rate was significantly higher in the ERA group than in the FET group.

Among the 408 RIF patients examined, 135 patients achieved successful live births, resulting in a live birth rate of 33.09%. Survival analysis results indicated that the live birth rate in the ERA group demonstrated a significant rise with an increasing number of transplantation cycles. Furthermore, the live birth rate in the ERA group consistently surpassed that of the FET group across all cycles, with a statistically significant difference (*p* < 0.05). These findings are presented in Figure 3.

### Kaplan Meier analysis of factors influencing live birth rates in patients with RIF

The Kaplan–Meier method was employed to analyze the factors that potentially influence the live birth rate of patients in each transfer cycle. The results of the study indicated that several variables, including AMH levels, the number of failed transfers, the number of embryos transferred, and the presence of good quality embryos for transfer, affected the patients’ live birth rate, with a statistically significant difference (*p* < 0.05). These findings are presented in Table 4.

**Table 4:** Kaplan–Meier analysis of live birth rates by transplantation cycle.

Characteristics	Number of people (*n* = 408)	Number of live birth (*n* = 135)	Median	*X* ^2^	*p*-value
Group					
ERA	204	75	7	2.491	0.115
FET	204	60	7		
Female age					
<35	264	93	7	1.659	0.106
≥35	144	42	7		
BMI					
Normal	341	112	7	2.036	0.175
Overweight/Obesity	67	23	5		
AFC					
Normal	148	57	7	3.014	0.096
Anomalies	260	78	7		
AMH					
Normal	148	82	7	5.374	0.020
Anomalies	260	53	5		
FSH					
Normal	306	100	7	2.084	0.172
Anomalies	102	35	5		
No. of previous implantation failure					
≤3	346	119	7	37.928	<0.001
>3	62	16	7		
Endometrial thickness					
Normal	232	82	7	1.258	0.162
Anomalies	176	53	5		
No. of embryos transferred					
1	166	38	7	9.400	0.002
2	242	97	5		
Embryo Quality					
Cycles with high-quality embryos	265	100	7	3.915	0.048
Cycles without high-quality embryos	143	35	6		

### Analysis of factors affecting live birth rates in patients with RIF using COX regression model

Table 5 presents the results of COX regression modeling, which aimed to analyze factors that may influence the live birth rate in patients with RIF. The findings revealed that patients who had two embryos transferred were more likely to achieve a successful live birth compared to those with a single embryo transfer (*HR* = 1.845, 95% *CI* 1.170–2.910, *p* < 0.05). Additionally, patients who received a high-quality embryo transfer had a higher likelihood of achieving a successful live birth compared to those without a high-quality embryo transfer (*HR* = 1.110, 95% *CI* 1.108–1.428, *p* < 0.05).

**Table 5:** COX regression analysis of patients’ live birth rates by transplant cycle.

Characteristics	*β*	*SE*	*wald*	*HR*	95%*CI*	*p*-value
Group						
ERA	-	-	-	1	-	-
FET	−0.306	0.285	2.722	0.736	0.512–1.256	0.106
Female age	0.013	0.026	0.242	1.013	0.962–1.066	0.623
BMI	0.017	0.035	0.237	1.017	0.962–1.066	0.627
AFC	−0.015	0.015	0.98	0.985	0.956–1.015	0.322
AMH	0.058	0.038	2.26	1.059	0.983–1.142	0.133
FSH	−0.008	0.05	0.027	1.008	0.914–1.112	0.87
No. of previous implantation failure						
≤3	-	-	-	1	-	-
>3	−2.903	0.548	28.096	0.055	0.019–0.160	<0.001
Endometrial thickness	0.044	0.055	0.64	1.045	0.938–1.164	0.424
No. of embryos transferred						
1	-	-	-	1	-	-
2	0.612	0.233	6.936	1.845	1.170–2.910	0.008
Embryo Quality						
Cycles without high-quality embryos	-	-	-	1	-	-
Cycles with high-quality embryos	0.104	0.129	0.659	1.11	1.108–1.428	0.417

## Discussion

The results of the study showed that the clinical pregnancy and live birth rates in the ERA group were 56.86 and 36.76%, respectively. Furthermore, the results of the study showed that the cumulative clinical pregnancy rate and cumulative live birth rate were higher in the ERA group than in the FET group, which is consistent with previous studies (11, 12). The physiological importance of the endometrium during pregnancy has received much attention in the field of reproductive medicine over the last few decades (13). ERA can help identify an appropriate window of implantation (WOI) for patients with repeated embryo implantation failure by analyzing gene expression, thereby increasing their chances of successful pregnancy. According to research findings, approximately 50% of failed pregnancies occur at a preclinical stage and may be due to chemical pregnancies or embryo implantation failure. (14). As it is well known, successful pregnancy and live birth are closely related to embryo quality, endometrial receptivity, and the synergy between the two. Even if patients undergo multiple transfers of high-quality embryos, including triploid blastocyst, they may still fail to achieve successful pregnancy, and therefore the treatment process needs to take into consideration factors related to endometrial receptivity. In such cases, further diagnosis and treatment may be necessary to ensure the adequacy of the endometrium and improve the chances of successful pregnancy and live birth. ERA is one of the most successful clinical applications for the diagnosis of endometrial receptivity, and it is one of the most successful clinical applications for the diagnosis of endometrial receptivity based on transcriptome analysis (15). ERA is effective in increasing pregnancy rates and the rate of live birth in patients treated with IVF-ET. However, in this study, although the cumulative live birth rate in the ERA group was higher than that in the FET group, the difference between the two groups was not statistically significant. This may be due to reasons such as insufficient sample size, and more randomized multicenter clinical trials need to be conducted to verify this in the future.

In this study, the clinical pregnancy rate of 50.58% and the live birth rate of 34.39% in the group with ≤3 embryo implantation failures were 30% (*p* < 0.05) and 25.81% (*p* < 0.05) higher than those in the group with >3 embryo implantation failures, respectively. Further multifactorial analysis showed that the group with >3 failed embryo implantations was 0.058 and 0.055 times more likely to have a successful pregnancy and live birth than the group with ≤3 failed embryo implantations, emphasizing the detrimental impact of multiple failed embryo implantations on successful pregnancy and live birth, and highlighting the importance of personalized treatment and careful consideration for these patients (16). This suggests that the pregnancy rate and the live birth rate are related to the number of previous implantation failures, and that as the number of failed embryo transfers increases in infertility patients, the likelihood of achieving a successful pregnancy and live birth decreases, which is similar to previous studies (17). This may be due to the fact that the higher the number of previous failed embryo transfers, the higher the medical costs and the greater the psychological strain on the patient. In addition, as the number of failures increases, the more likely it is that the patient will become infected and damaged in the uterus during the course of treatment, or it may be that the patient’s own embryonic and endometrial environment does not meet the conditions for a successful pregnancy (17). For patients with multiple failed embryo implantations, it is possible for them to experience psychological pressure and feelings of depression. Therefore, doctors should take this into consideration when devising treatment plans. Measures such as psychological counseling, supportive therapy, and providing family and social support should be implemented to alleviate the patients’ psychological stress and encourage them to maintain an optimistic and resilient attitude. At the same time, doctors should analyze the reasons for each failed implantation one by one and discuss with the patients how to develop targeted treatment plans to address these issues. By doing so, solutions can be found to prevent similar problems from recurring and improve the patients’ chances of successful pregnancy rate as well as the live birth rate.

The results of the current study showed that patients who had two embryos transferred during the course of treatment were 1.357 times more likely to have a successful pregnancy than those who had a single embryo transfer, and 1.845 times more likely to have a successful live birth than those who had a single embryo transfer. Some studies indicated that moderately increasing the number of embryos transferred in each cycle (up to three or four) can significantly improve the pregnancy rate and the success rate of IVF-ET. However, it is important to be cautious as this approach also increases the risk of multiple pregnancies, posing a threat to a woman’s pregnancy and life such as preterm birth and low birth weight. Therefore, when devising treatment plans, doctors and patients should communicate thoroughly and consider factors such as medical condition, reproductive history, and age to develop personalized transfer strategies that maximize treatment effectiveness and ensure safety during conception. (18). If a woman is not fit enough for a multiple pregnancy, the embryo may stop growing due to malnutrition or the baby may be born with a congenital defect such as mental retardation or low birth weight if effective measures are not taken (19). To promote embryo development and improve clinical pregnancy rates and live birth rates of patients, a comprehensive analysis from a medical perspective is needed. This involves conducting comprehensive evaluations of one’s own physical condition through clinical examinations, laboratory tests, etc. Under the guidance of professional doctors, we can determine the most suitable transfer strategy, including deciding on one-time transfers or multiple transfers because each individual’s physiological condition and embryo quality are different. Therefore, personalized treatment plans are crucial (20).

The current study also found that patients who had quality embryos transferred during treatment were 1.917 times more likely to have a successful pregnancy than those who did not have quality embryos transferred during treatment. It has been found that the most appropriate method to minimize the incidence and risks associated with multiple pregnancies is single embryo transfer (19). Globally, there is an increasing trend for patients to use single embryo transfer, but recent large-scale data also suggest that transfer of more than one embryo remains common in clinical practice (21). There is growing evidence that information exchange occurs between the embryo and the endometrium during implantation (22). The endometrium is not only an important site for implantation, but it also has sensor characteristics for assessing the quality of the blastocyst. It can receive signals about whether the blastocyst development is normal and translate these signals into acceptance or rejection responses in the endometrium. Poor-quality blastocysts may send abnormal and harmful signals to the endometrium due to inadequate nutrient supply, leading to rejection responses in the endometrium. These observations suggest that poor quality embryos may have a negative effect on endometrial acceptance (23). It has also been found that the addition of low-quality embryos to RIF patients alongside the transfer of high-quality embryos facilitates live births and multiple births (24). Preventive measures and treatment strategies such as ovarian protection, restoration of ovarian function, and environmental regulation are helpful in maintaining good communication between the blastocyst and the endometrium, improving successful pregnancy rates and reproductive outcomes for patients. Therefore, appropriate and targeted treatment protocols should be adopted in clinical practice to improve the pregnancy rate and live birth rate of patients.

In this study, we only included patients who underwent embryo transfer at the blastocyst stage. It is worth noting that transferring embryos at the blastocyst stage is physiologically more suitable as it closely mimics natural implantation timing, thereby improving the success rate of implantation. However, compared to fresh cleavage-stage embryos, there is relatively less clinical evidence regarding blastocyst transfer (25). Therefore, more research is needed in the future to further explore the effects and influencing factors of blastocyst transfer in order to provide positive guidance for clinical practice，and more work needs to be done in the future to understand the mechanisms, and larger populations and more scientific protocols are needed to assess the factors influencing pregnancy and live birth.

This study has a number of limitations. (1) The study was a retrospective cohort analysis, which introduces more bias than a prospective clinical cohort trial. (2) The sample size of the study was relatively small. However, we matched propensity scores between the two data sets so that there were no significant differences in baseline characteristics between the two groups, improving the scientific validity and reliability of the study results.

## Conclusion

In this study of patients with RIF treated with IVF-ET, we found that patients without ERA, >3 previous implantation failures, single embryo transfer, and no transfer of quality embryos were all predictors for clinical pregnancy in patients with RIF. having >3 previous implantation failures and using single embryo transfer were predictors for live birth in patients with RIF.

## Data availability statement

The raw data supporting the conclusions of this article will be made available by the authors, without undue reservation.

## Ethics statement

The studies involving humans were approved by the Ethics Review Committee of Chengdu Xi’nan Gynecology Hospital. Signed informed consent was obtained from all participants. The studies were conducted in accordance with the local legislation and institutional requirements. The participants provided their written informed consent to participate in this study. Written informed consent was obtained from the individual(s) for the publication of any potentially identifiable images or data included in this article.

## Author contributions

YJ: Writing – review & editing, Writing – original draft, Visualization, Supervision, Software, Resources, Methodology, Investigation, Funding acquisition, Formal analysis, Data curation, Conceptualization. ZA: Writing – review & editing, Writing – original draft, Visualization, Software, Resources, Methodology, Investigation, Formal analysis, Data curation. XZ: Writing – original draft, Data curation, Methodology, Formal analysis. ZC: Writing – original draft, Validation, Methodology. AP: Writing – original draft, Validation. ST: Writing – review & editing, Supervision, Data curation, Conceptualization. QZ: Writing – original draft, Methodology, Data curation, Conceptualization.
